# Couple concordance related to ITN use in Malawi

**DOI:** 10.1186/s12936-026-05877-1

**Published:** 2026-03-23

**Authors:** Bolanle Olapeju, Michael Bride, Anna Passaniti, Edson Dembo, Michael Kayange, Austin Gumbo, Taonga Mafuleka, Nyanyiwe Masingi Mbeye, Jennifer Boyle, Angela Chitsime, Alvin Chisambi

**Affiliations:** 1 Department of Preventive Medicine and Biostatistics, Uniformed Services University of the Health Sciences, Bethesda, MD USA; 2 Nuffield Department of Primary Care Health Sciences, University of Oxford, Oxford, UK; 3 Breakthrough ACTION, Johns Hopkins Center for Communication Programs, Baltimore, MD USA; 4 Malaria Branch, U.S. Centers for Disease Control and Prevention, Lilongwe, Malawi; 5 Ministry of Health, Lilongwe, Malawi; 6 Evidence Informed Decision-Making Centre, Department of Epidemiology and Biostatistics, Kamuzu University of Health Sciences, Lilongwe, Malawi

## Abstract

**Introduction:**

The shift to universal insecticide-treated nets (ITN) coverage in Malawi creates opportunities for establishing effective social and behavior change (SBC) interventions which target general populations, including couples. However, couple concordance in ITN use has not been well analyzed. We explored couple dynamics and concordance related to ITN use in Malawi to inform the potential role of couple-centered SBC approaches to improve consistent ITN use.

**Methods:**

The analysis focuses on 1213 male–female couples or dyads, a subset of a nationally representative survey. Key variables for both members of the couple included consistent net use and ITN Ideation, the combination of cognitive, social, and emotional factors related to a behavior measured as a composite score of several psychosocial factors. We assessed concordance (level of agreement) between men and women in the couple using Cohen’s Kappa statistic (K). Crude and adjusted logistic regressions identified factors associated with couples’ concordance in ITN ideation and consistent net use, adjusting for household and couple characteristics.

**Results:**

Over one-half (54%) of couple dyads reported consistent ITN use among both partners (k = 0.81). Factors significantly associated with couples’ concordance in high ITN ideation included residence in Central (adjusted odds ratio [aOR]: 2.27: 95% confidence interval [CI] 1.59–3.25) or Southern (aOR: 2.35; 95% CI 1.42–3.91) regions of Malawi compared to the North; wealthier households (aOR: 2.05; 95% CI 1.20–3.51); and having a primary education (aOR: 1.59; 95% CI 1.15–2.20). The most important couple-level factor associated with concordance in net use was concordance in ITN ideation (aOR: 1.66; 95% CI 1.08–2.55).

**Discussion:**

Our study demonstrated couples are likely either to jointly use or not use ITNs consistently, suggesting a couples’ approach may be worth exploring when designing and implementing SBC programs to promote consistent ITN use. This may necessitate addressing norms regarding couples’ use of ITNs to encourage discussion of malaria prevention and ITN use among couples.

**Supplementary Information:**

The online version contains supplementary material available at 10.1186/s12936-026-05877-1.

## Introduction

### Background

Malaria remains a significant health challenge in Malawi, with over four million cases in 2020 and over six million cases reported in 2024 [1]. The Ministry of Health’s Malaria Strategic Plan to reduce malaria incidence by at least 50 percent by 2022 has not yet been achieved [2]. However, opportunities persist with the current 2023–2030 plan to eliminate malaria by 2030 using several strategies including the use of insecticide-treated bed nets (ITN) for malaria prevention [3]. The World Health Organization (WHO) recommends that everyone in a malaria endemic area should sleep under an ITN every night and all through the night [4]. In line with this recommendation, the Government of Malawi provides regular ITN distributions, alongside other malaria prevention strategies in pregnancy, testing, and treatment services, at no cost for all Malawians [5].

National surveys show that consistent ITN use is generally high among those with access to nets [5, 6]. However, ITN use typically fluctuates with mass distribution campaigns and weather patterns [7], declining with lack of perceived severity of malaria [8], concerns about ITN safety [9] and net wear and tear [10]. Within households with nets specifically, ITN use was also higher among females compared to males [11] and highest among priority household members – pregnant women and children under five [12]. But the intra-household dynamics that drive consistent use among the general adult population are not well understood. This could include the size and composition of the household and household members’ decision-making, relative power and culture related factors influencing net use [13, 14] 

The shift to universal ITN coverage in Malawi presents an opportunity to look beyond individual prioritization and examine the interrelatedness of household members. The National Malaria Strategy and Communication Strategy aims to increase ITN use by targeting barriers to net use among (1) heads of household, (2) women of childbearing age, (3) adult patients, and (4) caretakers of sick children [9]. Secondary and tertiary audiences include husbands and other men in the community, older women in households, community leaders, health workers, and relevant government departments [5]. To date, couples have been underexplored as a target audience in Malawi and couple concordance- the degree of similarity in health status or behaviors between partners in a couple [15] related to ITN use is not well known. Understanding how partners agree or disagree on ITN related behaviors and outcomes provides a critical lens to understand and improve consistent ITN use within households.

Couple dynamics may play an important role in culturally accepted sleeping patterns and the acceptability or use of ITNs [16–18]. With a couple’s approach, the couple, not the individual, is the unit of interest. In Mozambique, consistent net use among couples was linked with partners sleeping together and shared decisions regarding net use was described as a sign of a harmonious household [19]. In Nigeria, couple-level factors involving parents increased ITN use among children under five included monogamy, interspousal discussion and concordance regarding their children using ITNs [20]. In Tanzania, decisions about ITN use, care, and repair were made primarily by women [16]. By tailoring malaria ITN use around couples and focusing on both partners, interventions might improve overall consistent bed net use and reduce the burden of malaria infections. However, to the best of the authors’ knowledge, there is no known research on couple concordance related to ITN use in Malawi.

### Conceptual frameworks

This study employs the ideation framework, a communication and behavior change model that highlights cognitive, emotional, and social factors influencing behavior change [21]. The underlying premise is that ideation includes the degree to which people (1) know about; (2) have positive attitudes about; (3) have a good feeling about; and (4) have spoken to others about a behavior. People are more likely to engage in a behavior if they have a high level of ideation [22].

**Fig. 1:**
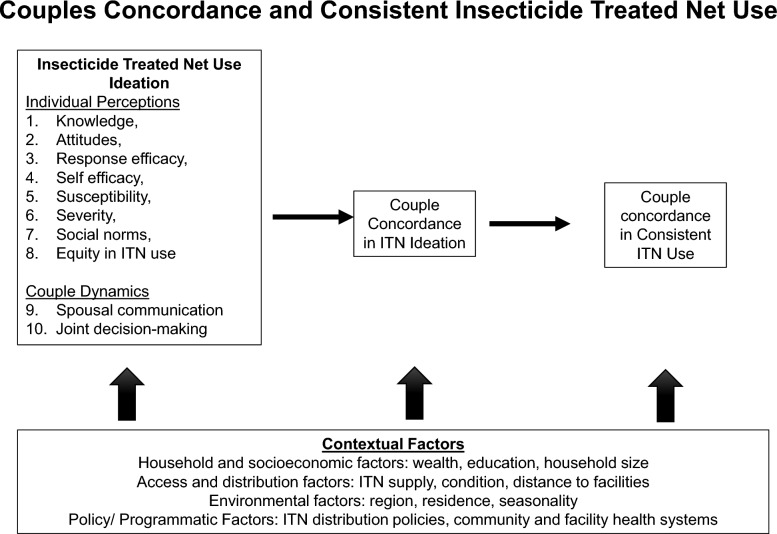
Study Conceptual Framework

The study conceptual framework (Fig. 1) posits that individual ideational factors shape each partner’s malaria prevention mindset. Through intra-couple communication and joint decision-making, partners develop ideational concordance, which strengthens shared behavior—consistent ITN use. The framework also highlights the role of context and social determinants of health as critical factors impacting individual- and couple- level ideation and behavior. Thus, using this framework, couples-focused social and behavior change interventions may amplify malaria prevention outcomes by targeting ideation alignment within couples. This framework has been widely used in malaria prevention and treatment including consistent ITN use [23], care-seeking for fever [24] and treatment adherence [25].

### Study aims and rationale

This study, conducted in partnership with the Malawi National Malaria Control Programme, investigates the sociological factors and ideational determinants that influence how couples jointly engage in consistent ITN use. By understanding the level of agreement between partners regarding ITN use and ideation, study findings can inform relevant programmatic and policy recommendations on the design of specific communication and behavior change strategies tailored to couples that on both partners. Study findings can also inform the extent to which a couple-centered approach could improve consistent net use and ultimately reduce the malaria burden in Malawi.

## Methods

### Study design and population

This analysis relies on data collected from the U.S. President’s Malaria Initiative-funded Malaria Behavior Survey (MBS) implemented by the Johns Hopkins Center for Communication Programs’ Breakthrough ACTION project in 2021 [6]. The MBS was a nationally representative cross-sectional study conducted in collaboration with the NMCP of the Malawi MOH that assessed ideational determinants and psychosocial factors of key malaria-related behaviors [6].

The Malawi MBS team randomly selected 62 enumeration areas across all three regions of Malawi and then, to meet the study sample size of 3906 households, randomly selected and interviewed 21 households from each enumeration area. A household head/representative and all women aged 15–49 was interviewed in every household. In addition, a male aged 18–59 years who was the spouse/partner of an eligible woman was interviewed in every third household. Data was collected between 26 May 2021 and 1 July 2021. The final sample included 3862 households, including 4181 women and 1304 men interviewed. Complete data on all study variables for men and women in the same household was available for 1213 women and their partners. Thus, this manuscript focuses on 1213 male–female couples or dyads.

### Power calculation

The sample size of 1213 couples provides 80% power to detect a 5% margin of error in the estimate of concordance in the consistent use of net among couples assuming a prevalence of 50% for maximum variability and an alpha (Type 1 error rate) of 0.05.

### Ethical considerations

An ethical review of the MBS study was conducted and approved by both the institutional review boards from the Johns Hopkins Bloomberg School of Public Health (IRB No. 15731) and the Malawi National Health Sciences Research Committee (NHSRC No. 19/11/2447). Prior to participating in the survey, all respondents provided written consent. For female minors aged 15–17 parents provided permission after which the minors provided assent.

### Data collection

Surveys were conducted face-to-face with the heads of household, eligible women, and an eligible man (in one-third of households). The household questionnaires administered to the household heads covered household characteristics, ownership of assets, and a roster of all bed nets (used) in the house. Both women’s and men’s questionnaires included modules assessing net use, care, and disposal; perceptions of health services; ideational factors related to malaria behaviors, and exposure to malaria-related messages. Of note, women’s questionnaires also explored antenatal care and receipt of intermittent preventive treatment of malaria for pregnant women among women who had a live birth within the past two years, as well as receipt of appropriate treatment for children who had a fever in the past two weeks.

### Key variables

The key outcomes of interest were consistent net use and ITN ideation. Consistent net use was defined as sleeping under a net every night for the past seven days preceding the survey and was self-reported. This aligns with the NMCP communication during ITN distribution that residents sleep under a net every night, all night, all year long [5]. ITN Ideation was a composite score comprised of the following ideational factors: correct knowledge related to ITNs, favorable attitudes about ITNs, perceived response efficacy of ITNs, perceived self-efficacy to use ITNs, perceived severity of malaria, perceived susceptibility of malaria, recent couples discussion of malaria, joint decision making related to malaria, supportive community norms towards ITNs, and perceived equity related to sex and ITNs. Further breakdown of the questions and responses are included in Supplemental Table 1.

**Table 1:** Description of Study Population (N = 1213 couples)

Characteristics	Northern n (%)	Central n (%)	Southern n (%)	Total n (%)	P-value
Number of couples	402 (35%)	390 (31%)	421 (34%)	1,213 (100%)	
Rural residence	51 (29%)	64 (45%)	58 (26%)	173 (100%)	0.300
Wealth quintile					
Poorest	42 (23%)	106 (55%)	53 (21%)	201 (100%)	< 0.001
Poorer	69 (33%)	84 (34%)	95 (34%)	248 (100%)	
Middle	93 (40%)	67 (24%)	103 (36%)	263 (100%)	
Richer	84 (35%)	67 (23%)	90 (42%)	241 (100%)	
Richest	114 (41%)	66 (24%)	80 (34%)	260 (100%)	
Near* a health facility	207 (32%)	188 (29%)	236 (39%)	631 (100%)	0.079
≥ 4 members	295 (37%)	252 (28%)	283 (35%)	830 (100%)	0.024
≥ 1 ITN/2 people	68 (24%)	55 (27%)	92 (49%)	215 (100%)	0.014
Couple has ≥ primary education	156 (36%)	121 (30%)	121 (34%)	398 (100%)	0.006
Couple is ≤ 30 years old	123 (35%)	149 (37%)	137 (28%)	409 (100%)	0.06
Couple is Christian	387 (40%)	343 (31%)	301 (29%)	1,031 (100%)	< 0.001
Couple has a child < 5 years	268 (37%)	243 (30%)	248 (32%)	759 (100%)	0.07
Couple is currently pregnant	30 (24%)	42 (37%)	51 (39%)	123 (100%)	

^*^Near is defined as < 5 km, 30 min by foot, or 10 min by car

Contextual variables included in the analysis were household-level variables such as residence (urban or rural) household wealth quintile (based on ownership of household assets), household size (≤ 4 people versus not), the household’s proximity to a health facility (under 5 km, 30 min by foot, or 10 min by car) and adequate supply of ITNs in the household (at least one ITN per two people in the household). Couple-level contextual variables included education (both man and woman completed primary education versus not), region (Northern, Central, or Southern), age (both man and woman aged 30 years or less versus not), religion (both man and woman were Christian versus not), presence of young children (the couple had a child(ren) under 5 years old versus not), whether they had an adequate supply of ITNs in their household (measured at one ITN per two people or not) and whether the woman was pregnant or not.

### Data analysis

All ideational factors were dichotomized into 1 and 0, based on if all responses were correct versus not. The overall ITN ideation score was a composite sum of all 10 dichotomized variables and thus ranged from 0 to 10 points with a median of 6 points. The score was dichotomized into high (> 5 points) ideation and low (≤ 5 points) ideation based on the median score.

Concordance between men and women’s reports of ITN ideational factors, overall ITN ideation, and consistent net use was assessed using Cohen’s Kappa (K) for categorical variables and Lin’s concordance correlation coefficient (CCC) for continuous variables. Using the criterion proposed by Landis and Koch, correlation coefficients were categorized according to the strength of agreement: Poor (< 0.00), Slight (0.00–0.20), Fair (0.21–0.40), Moderate (0.41–0.60), Substantial (0.61–0.80), and Almost Perfect (0.81–1.00) [26].

Kappa was used to explore partner concordance around the key variables of interest as well as the score of all ideational determinants and consistent ITN use. Crude and adjusted logistic regressions were used to determine factors associated with couple concordance in ITN ideation and consistent net use. Adjusted models controlled for urban versus rural residence, wealth quintile, region, age, religion, whether the couple were pregnant, had a child under 5, adequate ITN supply, and ideational factors including correct knowledge related to ITNs, favorable attitudes, perceived response efficacy, perceived self-efficacy, perceived severity, perceived susceptibility, recent discussion of malaria, joint decision making related to malaria, and supportive community norms related to ITNs and equity.

Researchers performed data management and analysis using Stata version 16 (Stata Corporation, College Station, TX, USA) and Excel 2016 (Microsoft Corp, Seattle, WA, USA). The data were weighted using the svyset command in Stata to make the data representative of the study population.

## Results

### Description of study population

Table 1 describes the demographic characteristics of the study population at the household- and couple-levels. Most couples lived in rural areas (86%), were Christian (85%), lived in households with more than four members (69%), or had a child under five years old (68%). On the other hand, less than one-half of couples lived in households close to a health facility (48%) and only one-third of couples had at least a primary education (33%) or were younger than 30 years old (34%). About one-fifth of couples lived in households with enough ITNs (18%) or in the lowest wealth quintile (17%). Of note, most couples’ demographic characteristics were significantly associated with their region of residence, including wealth quintile, education, religion, and ownership of enough ITNs.

### Couple concordance in ITN ideation and consistent use

Table 2 summarizes couple concordance in ITN ideation and consistent net use. In general, more prevalent ideational factors included correct knowledge of ITN use, perceived self-efficacy to use ITNs, susceptibility to malaria, severity of malaria and perceived equality related to sex and malaria. The least prevalent ideational factor was recent inter-partner communication related to malaria. Ideational factors which were identical among men and women included perceived susceptibility (85%) and perceived severity (92%) while joint decision-making, supportive community norms, and perceived equity related to ITNs and sex differed between men and women by just one percentage point across these factors. Favorable attitudes and perceived response efficacy of ITNs varied by five or more percentage points across men and women while perceived self-efficacy of ITNs varied considerably among men (91%) and women (57%).

**Table 2:** Couple Concordance in ITN Ideation and Consistent Use (N = 213)

Factor	% Men (N = 1213)	% Women (N = 1213)	% Couple (N = 1213)	% Couple agreement	Kappa statistic
ITN Ideation					
1. Correct knowledge related to ITNs	89	91	83	85	0.21
2. Favorable attitudes towards ITNs	52	47	31	62	0.24
3. Perceived response efficacy of ITNs	64	61	45	62	0.22
4. Perceived self-efficacy of ITNs	90	61	54	58	0.04
5. Perceived susceptibility of malaria	86	86	76	100	1.00
6. Perceived severity of Malaria	89	90	86	93	0.50
7. Supportive community norms towards ITNs	56	57	41	67	0.33
8. Perceived equity related to ITNs and sex	99	98	97	97	0.04
9. Recent discussion of malaria	30	34	16	75	0.42
10. Joint decision-making related to malaria	58	54	43	73	0.45
High ITN ideation^a^	85	75	68	76	0.27
Consistent ITN use	56	59	54	91	0.81

High ITN ideation defined as a composite score of > 5 points based on the median score.

Across all ideational factors, the kappa statistic ranged between 0.04 and 0.5 or less indicating poor to moderate agreement. Most men (86%), women (75%) and couples (68%) had an overall high level of ideation. However, the kappa statistic was 0.27 indicating fair agreement. In comparison, 57% of men, 61% of women and 54% of couples reported consistent net use, and the kappa statistic was 0.81 indicating substantial to almost perfect agreement.

**Fig. 2:**
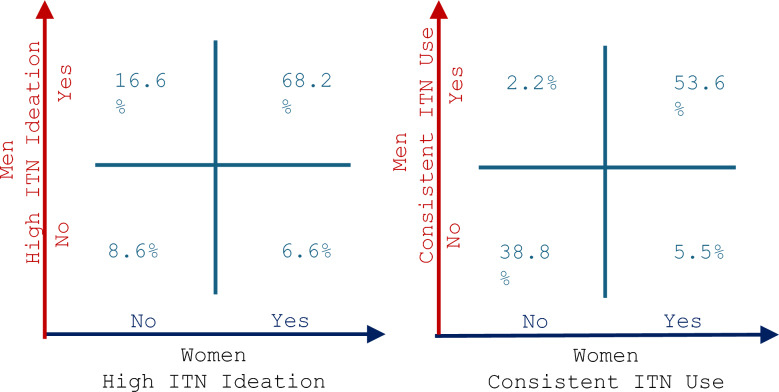
Distribution of ITN ideation and consistent net use among men and women partners

Figure 2 shows the distribution of ITN ideation and consistent net use among men and women partners. In 68% of couples, ITN ideation was high for both men and women while in 9% of couples, ITN ideation was low in both men and women. For 17% of couples, ITN ideation was high in only the men while in 7%, ITN ideation was high in only the women. On the other hand, 54% of couples reported consistent ITN use among both men and women. In 39% of couples, both men and women did not use ITNs consistently. In just 2% of couples, only the men reported consistent net use and in 6% only the women reported consistent net use.

### Factors associated with couple concordance in high ITN ideation

As highlighted in Table 3, household-level factors significantly associated with couple’s concordance in high ITN ideation included residence in Central (adjusted odds ratio [aOR]: 2.27; 95% confidence interval [CI] 1.59–3.25) or Southern (aOR: 2.35; 95% CI 1.42–3.91) regions compared to the North and wealthier households compared to the least wealthy households (aOR: 2.05; 95% CI 1.20–3.51). The couple-level factors influencing concordance in ITN ideation was limited to primary education (aOR: 1.59; 95% CI 1.20–3.51).

**Table 3:** Factors predicting couple concordance in high ITN ideation (N = 1,213)

Couple characteristics	N (%) of couples	Odds of concordance in ITN ideation	
		Odds ratio (95% CI)	aOR (95% CI)
Region			
Northern (ref)	182 (43%)	1.00 (N/A)	1.00 (N/A)
Central	229 (58%)	1.72 (1.30–2.28)	2.27 (1.59–3.25)
Southern	235 (63%)	1.53 (1.16–2.01)	2.35 (1.42–3.91)
Residence			
Urban (ref)	559 (54%)	1.00 (N/A)	1.00 (N/A)
Rural	87 (53%)	0.87 (0.63–1.20)	0.62 (0.42–0.92)
Wealth quintile			
Poorest (ref)	94 (46%)	1.00 (N/A)	1.00 (N/A)
Poorer	115 (45%)	0.98 (0.68–1.43)	0.97 (0.62–1.51)
Middle	146 (53%)	1.42 (0.98–2.05)	1.41 (0.88–2.27)
Richer	145 (63%)	1.72 (1.18–2.51)	2.05 (1.20–3.51)
Richest	146 (61%)	1.46 (1.01–2.11)	1.67 (0.97–2.88)
Near^a^ a health facility			
No (ref)	288 (50%)	1.00 (N/A)	1.00 (N/A)
Yes	358 (58%)	1.34 (1.07–1.68)	1.15 (0.89–1.49)
Household size ≥ 4			
No (ref)	518 (52%)	1.00 (N/A)	1.00 (N/A)
Yes	128 (55%)	1.05 (0.82–1.33)	1.18 (0.82–1.71)
ITN supply enough			
No (ref)	518 (51%)	1.00 (N/A)	1.00 (N/A)
Yes	128 (67%)	1.36 (1.01–1.84)	1.42 (0.97–2.08)
Primary education			
No (ref)	392 (49%)	1.00 (N/A)	1.00 (N/A)
Yes	254 (64%)	1.90 (1.49–2.44)	1.59 (1.15–2.20)
Age (< 30)			
No (ref)	429 (55%)	1.00 (N/A)	1.00 (N/A)
Yes	217 (52%)	0.99 (0.78–1.25)	1.03 (0.74–1.45)
Christian religion			
No (ref)	93 (50%)	1.00 (N/A)	1.00 (N/A)
Yes	553 (55%)	1.11 (0.81–1.52)	1.38 (0.89–2.12)
Child < 5 years			
No (ref)	251 (55%)	1.00 (N/A)	1.00 (N/A)
Yes	395 (54%)	0.88 (0.69–1.11)	1.03 (0.77–1.37)
Pregnant			
No (ref)	571 (54%)	1.00 (N/A)	1.00 (N/A)
Yes	75 (60%)	1.42 (0.97–2.08)	1.35 (0.88–2.09)

^a^Near is defined as < 5 km, 30 min by foot, or 10 min by car

*ref* reference, *N/A* Not Applicable

### Factors predicting couple concordance in consistent net use

As shown in Table 4, among couples residing in households with at least one ITN, significant household-level factors included residence in Central (aOR: 2.23; 95% CI 1.32–3.78) or Southern (aOR: 2.32; 95% CI 1.27–4.26) regions compared to the North; residence in urban (aOR: 0.44; 95% CI 0.24–0.78) compared to rural areas; and having enough ITNs in the household (aOR: 2.43; 95% CI 1.39–4.23). The most important couple-level factor associated with concordance in net use was concordance in ITN ideation (aOR: 1.66; 95% CI 1.08–2.55). None of the other couple-level factors were associated with consistent net use.

**Table 4:** Factors predicting couple concordance in consistent net use among couples with ITNs (N = 774)

Characteristics	N (%) of Couples	Odds of concordance in consistent ITN use	
		OR (95% CI)	aOR (95% CI)
High ideation			
No (ref)	262 (77%)	1.00 (N/A)	1.00 (N/A)
Yes	354 (86%)	1.42 (1.00–2.02)	1.66 (1.08–2.55)
Region			
Northern (ref)	177 (74%)	1.00 (N/A)	1.00 (N/A)
Central	193 (85%)	1.78 (1.15–2.76)	2.23 (1.32–3.78)
Southern	246 (87%)	1.86 (1.23–2.82)	2.32 (1.27–4.26)
Residence			
Urban (ref)	533 (84%)	1.00 (N/A)	1.00 (N/A)
Rural	83 (71%)	0.59 (0.38–0.92)	0.44 (0.24–0.78)
Wealth quintile			
Poorest (ref)	84 (81%)	1.00 (N/A)	1.00 (N/A)
Poorer	125 (87%)	1.68 (0.90–3.14)	1.74 (0.83–3.65)
Middle	133 (78%)	1.29 (0.72–2.31)	0.99 (0.47–2.09)
Richer	134 (83%)	1.34 (0.74–2.41)	1.30 (0.62–2.69)
Richest	140 (82%)	0.94 (0.54–1.64)	0.99 (0.48–2.08)
Near^a^ a health facility			
No (ref)	266 (84%)	1.00 (N/A)	1.00 (N/A)
Yes	350 (81%)	0.83 (0.58–1.18)	0.75 (0.47–1.19)
Household size ≥ 4			
No (ref)	192 (84%)	1.00 (N/A)	1.00 (N/A)
Yes	424 (81%)	0.83 (0.56–1.22)	0.76 (0.41–1.41)
ITN supply enough			
No (ref)	427 (79%)	1.00 (N/A)	1.00 (N/A)
Yes	189 (90%)	2.25 (1.43–3.54)	2.43 (1.39–4.23)
Primary education			
No (ref)	383 (81%)	1.00 (N/A)	1.00 (N/A)
Yes	233 (83%)	1.05 (0.73–1.51)	1.19 (0.72–1.96)
Age (< 30)			
No (ref)	417 (82%)	1.00 (N/A)	1.00 (N/A)
Yes	199 (82%)	0.97 (0.67–1.41)	0.82 (0.47–1.41)
Christian religion			
No (ref)	91 (81%)	1.00 (N/A)	1.00 (N/A)
Yes	525 (82%)	0.98 (0.60–1.61)	1.52 (0.83–2.79)
Child < 5 years			
No (ref)	217 (83%)	1.00 (N/A)	1.00 (N/A)
Yes	399 (82%)	0.93 (0.64–1.34)	1.11 (0.70–1.78)
Pregnant			
No (ref)	552 (83%)	1.00 (N/A)	1.00 (N/A)
Yes	64 (79%)	0.85 (0.49–1.46)	0.55 (0.27–1.12)

^a^Near is defined as < 5 km, 30 min by foot, or 10 min by car

*ref* reference, *N/A* Not Applicable

## Discussion

Couples’ concordance has been well documented as a predictor of several health behaviors and outcomes including mental health, smoking cessation, family planning use, quality sleep and influenza vaccinations [27–29]. To the best of our knowledge, this is the first study to explore couples concordance and dynamics related to consistent ITN use. Using nationally representative data, this study examined how couple characteristics and differences relate to ITN ideation and consistent net use. The findings contribute to a deeper understanding of the interrelationships between individual and couple-level psychosocial factors and preventive health behaviors.

We found comparatively high levels of couple’s agreement in perceived severity and perceived susceptibility to malaria, and joint decision-making related to malaria but relatively low agreement on perceived self-efficacy to use ITNs. Although overall concordance in ITN ideation was modest, couples with higher concordance in ideation were significantly more likely to consistently use nets. The findings also revealed substantial concordance in consistent ITN use within couples.

These results have several programmatic implications. Findings suggest that considering couples as an audience group may support and facilitate consistent ITN use within households. Social and behavior change (SBC) messages may benefit from leveraging existing joint decision-making among couples and encouraging intra-household discussions about malaria prevention. Communication efforts could also build upon couples shared perceptions of malaria severity and susceptibility, while addressing gaps in self-efficacy and attitudes toward ITN use.

Traditional approaches targeting men and women separately in traditionally masculine (e.g., farming or sports) versus feminine (e.g., hospitals) spaces may be complemented by approaches that engage couples together. For instance, interpersonal or community engagement activities (e.g., couples discussion groups) and mass media content (e.g., radio or TV dramas featuring couples) may enhance dialogue and shared responsibility for malaria prevention. Evidence from Malawi indicates that community radio and interpersonal engagement are effective channels for malaria-related communication [30]; couple-focused messages may further strengthen these efforts.

The strong association between ITN supply and consistent use underscores the continued importance of ensuring households have sufficient access to nets prior to or alongside SBC interventions [22]. Ongoing efforts to ensure that ITN mass distribution campaigns occur every two to three years in Malawi, may benefit from routine distribution channels such as antenatal care, immunization or well-baby clinics, and school distributions, which have demonstrably boosted ITN coverage levels [31].

Findings also point to opportunities for research to better understand the contextual factors influencing couples’ ITN use, including decision-making, net maintenance, and gender norms. Qualitative or longitudinal studies could further elucidate how couples’ dynamics evolve and interact with behavior change over time. Research implications of our study findings also include the need for longitudinal studies to corroborate study findings and while addressing existing limitations in our cross-sectional study including potential recall bias and inability to demonstrate causality. Qualitative approaches may be needed to uncover existing community norms related to the couple's joint use of ITNs. While this study found that couples who had children or were pregnant did not have higher odds of ITN ideation on consistent ITN use, it would be interesting to explore other dimensions of decision making and ITN use among members of the household that are not couples, such as siblings.

Future studies should aim to better understand the context of couples’ ITN use, including hanging, folding, and care of nets, as well as decision-making dynamics, delineation of roles and responsibilities related to ITN acquisition, and use and care in the household. Such research should inform the human-centered design of interventions that ensure participation from couples in co-creating contextually relevant SBC solutions, including but not limited to behavioral economics or communication interventions.

Policy implications of study findings may include a preliminary data-driven recommendation to include couples as an audience group in the National Malaria Communication Strategy. Additional efforts may be required to guide the implementation of such a couples’ approach in the National Malaria Communication Strategy. Complementary efforts to promote the prioritization of couples, men and boys in ITN use communication campaigns should be explored. A review of current laws, regulations, or relevant policies may be instituted to ensure a supportive context for a multisectoral malaria strategy that addresses the needs and vulnerabilities of men, women, girls, and boys, thus ensuring the future well-being of couples.

In conclusion, this novel research explored and uncovered opportunities for the inclusion of a couples’ approach in the design and implementation of relevant contextually tailored multi-level solutions to address consistent ITN use in Malawi.

## Supplementary Information

Supplementary Material 1

## Data Availability

Technical appendix, statistical code, and datasets for this study are available upon request at the [USAID Development Data Library] (https:/data.usaid.gov/browse?limitTo = datasets).
