# Supplementary material for: Couple concordance related to ITN use in Malawi
Source: Malar J. 2026 Mar 23;25:189. doi: 10.1186/s12936-026-05877-1 (PMC13134269; doi:10.1186/s12936-026-05877-1)
Supplement: Supplementary file 1 — Supplementary Material 1 [file 12936_2026_5877_MOESM1_ESM.docx]

### Supplemental Table 1: Key Variables and Correct Responses

| **Variable** | **Correct Response** |
| --- | --- |
| ***Individual Perceptions*** |  |
| 1. *Correct knowledge related to ITNs* | |
| What causes malaria? | Mosquito bite |
| What are the things that people can do to stop them from getting malaria? Bed nets are a method of malaria prevention | Sleep under a mosquito net or ITN |
| 1. *Favorable attitudes towards ITNs* | |
| It is not easy to sleep under a net because every night you have to unfold it and cover the sleeping space | Disagree |
| I do not like sleeping under a mosquito net when the weather is too warm | Disagree |
| The smell of the insecticide makes it uncomfortable for me to sleep under a mosquito net | Disagree |
| Insecticide-treated nets do not pose a risk to one's health | Agree |
| 1. *Perceived response efficacy of ITNs* | |
| Mosquito nets prevent mosquito bites only when used on a bed | Disagree |
| The chances of getting malaria are the same whether or not one sleeps under a mosquito net | Disagree |
| Sleeping under a mosquito net every night is a good way to avoid getting malaria | Agree |
| 1. *Perceived self-efficacy to use ITNs* | |
| Sleep under a mosquito net for the entire night when there are lots of mosquitoes | Agree |
| Sleep under a mosquito net for the entire night when there are few mosquitoes | Agree |
| Sleep under a mosquito net every night of the year | Agree |
| 1. *Perceived susceptibility of malaria* | |
| Nearly every year, someone in this community gets a serious case of malaria | Agree |
| 1. *Perceived severity of malaria* | |
| Every case of malaria can potentially lead to death | Agree |
| 1. *Supportive community norms towards ITNs* | |
| Generally, among the people in your community who have nets, how many sleep under them every night? | At least half of the community |
| Generally, among the people in your community, how many people would call you names if they know that you sleep under a net every night? | Less than half of the community |
| 1. *Perceived equity related to ITNs and sex* | |
| When there aren’t enough nets, it is more important that male children sleep under the available nets rather than female children. | Disagree |
| When there aren’t enough nets, it is more important that female children sleep under the available nets rather than male children. | Disagree |
| ***Couple Dynamics*** |  |
| 1. *Interpersonal communication related to malaria* |  |
| In the last six months, did you talk about malaria with your spouse or partner? | Yes |
| 1. *Joint decision-making related to malaria* | |
| In your household, who usually makes decisions about what to do when you are sick? | Joint decision with spouse/partner |
| **Consistent ITN use** | |
| On average, how many nights in a week do you sleep under a mosquito net? | Every night |
